# Comparative effectiveness of treatments for recurrent *Clostridioides difficile* infection: a network meta-analysis of randomized controlled trials

**DOI:** 10.3389/fphar.2024.1430724

**Published:** 2024-10-17

**Authors:** Hong Duo, Yanwei Yang, Guqing Zhang, Yingxin Chen, Yumeng Cao, Linjie Luo, Huaqin Pan, Qifa Ye

**Affiliations:** ^1^ Hubei Key Laboratory of Medical Technology on Transplantation, National Quality Control Center for Donated Organ Procurement, Hubei Clinical Research Center for Natural Polymer Biological Liver, Hubei Engineering Center of Natural Polymer-Based Medical Materials, Zhongnan Hospital of Wuhan University, Institute of Hepatobiliary Diseases of Wuhan University, Transplant Center of Wuhan University, Wuhan, Hubei, China; ^2^ Department of Critical Care Medicine, Zhongnan Hospital of Wuhan University, Clinical Research Center of Hubei Critical Care Medicine, Wuhan, China; ^3^ Department of Respiratory and Critical Care Medicine, Zhongnan Hospital of Wuhan University, Wuhan, China; ^4^ Global Health Institute, School of Public Health, Xi’an Jiaotong University Health Science Center, Xi’an, China; ^5^ Department of Experimental Radiation Oncology and Surgical Oncology, The University of Texas MD Anderson Cancer Center, Houston, United States; ^6^ Zhongnan Hospital of Wuhan University, Institute of Hepatobiliary Diseases of Wuhan University, Transplantation Intensive Care Unit, Transplant Center of Wuhan University, Hubei Key Laboratory of Medical Technology on Transplantation, Wuhan, China

**Keywords:** Clostridioides difficile, infection, fecal microbiota transplantation, randomized controlled trials, network meta-analysis

## Abstract

**Background:**

Clostridioides difficile infection (CDI) is the most common cause of healthcare-associated infectious diarrhea. A major clinical challenge is recurrent CDI (rCDI) without effective standard drug-based therapy. Additionally, a comprehensive comparison of various therapy effectiveness in rCDI patients is still under investigation.

**Methods:**

A Bayesian network meta-analysis (NMA) of randomized control trials up to March 2024 was performed to investigate the efficacy of rCDI interventions.

**Results:**

Seventeen trials were included, comprising 4,148 CDI patients with ten interventions, including fecal microbiota transplantation (FMT) by lower gastrointestinal (LGI), FMT by upper gastrointestinal (UGI), Autologous FMT (AFMT), vancomycin + FMT, vancomycin, placebo, fidaxomicin, Vowst (SER109), Rebyota (RBX2660), and monoclonal antibody. NMA showed that FMT by LGI had the highest efficacy in treating rCDIs with an odds ratio (95% confidence interval) of 32.33 (4.03, 248.69) compared with placebo. FMT by UGI also showed high efficacy, whereas the efficacy comparison between FMT by LGI and UGI was not statistically significant (ORs) (95% CI), 1.72 (0.65, 5.21). The rankogram and surface under the cumulative ranking curve (SUCRA) also showed FMT by LGI ranked at the top and FMT by UGI ranked second in the curative effect.

**Conclusion:**

NMA demonstrates FMT’s significant efficacy in rCDI management, regardless of administration route (lower or upper gastrointestinal). Despite its significant benefits, FMT’s safety is a concern due to the lack of standardized FDAcompliant manufacturing and oversight. Microbiota-based therapies also exhibit potential. However, limited research mandates further clinical exploration. Antibiotics, in contrast, display comparatively reduced efficacy in rCDI, potentially linked to disruptions in native gut microflora balance.

**Systematic Review:**

https://www.crd.york.ac.uk/PROSPERO/display_record.php?RecordID=368435, Identifier CRD42022368435.

## 1 Introduction

*Clostridioides difficile* infection (CDI) is the most common cause of healthcare-associated infectious diarrhea, usually due to a side-effect of antibiotic therapy (Magill et al., 2014). The symptoms of CDI include diarrhea, fever, nausea, and stomach tenderness in most patients, but it can also cause severe or fatal complications, such as ileus or toxic megacolon (Guh and Kutty, 2018). It affects approximately 500,000 people in the United States, leading to about 30,000 deaths annually (Dubberke and Olsen, 2012; Feuerstadt, Theriault and Tillotson, 2023; Guh et al., 2020).

Since the 1970s, patients with CDI have been treated with metronidazole and vancomycin. For severe cases, vancomycin shows better efficacy than metronidazole, but vancomycin and metronidazole have been considered equally effective for mild-to-moderate patients (Zar et al., 2007). However, more than one-third of the patients who had already experienced a recurrence will relapse after receiving metronidazole or vancomycin treatment, while the relapse rate for patients who did not have a recurrence is typically 20%–30% (Polivkova, Krutova, Capek, Sykorova and Benes, 2021). The previous study shows that indigenous intestinal microbiota, which can protect against CDI, was suppressed by antibiotics, such as vancomycin, a broad-spectrum Gram-positive antimicrobial, and fidaxomicin, a narrow-spectrum but expensive antibiotic (Khoruts, Staley and Sadowsky, 2021). A major clinical challenge is recurrent CDI (rCDI). Around 35% of patients with rCDI will eventually relapse, and 60% of those patients will have multiple recurrences (Feuerstadt et al., 2023). In recent years, fecal microbiota transplantation (FMT), referring to the delivery of fecal floras from a healthy donor into the patient’s gastrointestinal tract, has emerged as a safe and effective therapy for rCDI (Kakihana et al., 2016). FMT was recommended for patients with rCDI by the British Society of Gastroenterology in 2018 (Mullish et al., 2018). In 2021, it was recommended for patients with severe and fulminant CDI (C. R. Kelly et al., 2021). FMT reshapes a healthy and diverse gut microbiome in CDI patients, restoring their resistance to *Clostridioides difficile* infection (Seekatz et al., 2014). However, there is controversy about the site of FMT is administered and that FMT is not without its dangers. A systematic review reported that diarrhea resolution rates were related to the administration site of FMT: stomach (81%), duodenum/jejunum (86%), cecum/ascending colon (93%), and distal colon (84%) (Cammarota et al., 2014). However, a new RCT found that both nasogastric tube (NGT) and colonoscopic administration are equally effective (Youngster et al., 2014). Based on the American College of Gastroenterology (ACG) guidelines, delivering FMT through colonoscopy or capsules to treat rCDI is better ^11^. A new meta-analysis shows that upper modalities were less effective than lower administration (Quraishi et al., 2017). Therefore, our objective is to identify an optimal administration site for FMT. Besides, donor fecal microbiota transplantation (DFMT) will lead to the risk of infection. Autologous fecal microbiota transplantation (AFMT) may reduce the risk of infection. However, an RCT reported that the effect of AFMT was poorer than DFMT (Kelly et al., 2016). Trials with manufactured human-derived microbiota (Rebyota: RBX2660) and purified Firmicutes spores (Vowst: SER109) that have recently been approved for clinical use by the U.S. Food and Drug Administration (FDA) as novel products are microbiota-based therapeutics for the treatment of rCDI, and another FDA approved product, a monoclonal antibody named bezlotoxumab (zinplava) against *Clostridioides difficile* toxin B (Khanna et al., 2022; Sims et al., 2023; Wilcox et al., 2017).

Network meta-analysis (NMA) is a meta-analysis in which multiple treatments are simultaneously analyzed using direct comparisons of interventions within randomized controlled trials (RCTs) and indirect comparisons across trials based on a common comparator, such as a placebo or some standard treatments (Caldwell, 2014; Caldwell, Ades and Higgins, 2005; Glenny et al., 2005; Higgins and Whitehead, 1996; Lu and Ades, 2004; Lumley, 2002). Compared to conventional pair-wise meta-analysis which yields only one pooled effect estimate, a network meta-analysis produces more than one pooled effect estimate, providing more evidence of comparative effectiveness that is valuable for clinical decision-making (Tonin, Rotta, Mendes and Pontarolo, 2017). However, most existing meta-analyses related to CDI merely focused on case series evidence instead of randomized controlled trials (RCTs) (Pomares Bascuñana, Veses and Sheth, 2021; Ramai et al., 2021; Tariq, Pardi, Bartlett and Khanna, 2019; Tixier et al., 2022), and there were several novel, relative RCTs were recently published (Feuerstadt et al., 2022; Jiang et al., 2018; Kao et al., 2017; Khanna et al., 2022; McGovern et al., 2021; I; Youngster et al., 2014). Therefore, we incorporated all the available published RCTs and conducted a network meta-analysis to investigate the optimal strategy for treating rCDI, mainly focusing on the route of FMT delivery and conventional and novel interventions, which provided clinical insights for curing rCDI.

## 2 Materials and methods

We used the Preferred Reporting Items for Systematic Reviews and Meta-Analyses (PRISMA) and the extension statement for NMA guidelines (PRISM-NMA) to conduct this network meta-analysis (Hutton et al., 2015; Page et al., 2021) (Supplementary Table S1 for PRISMA-NMA Checklist). This systematic review and network meta-analyses protocol was registered with PROSPERO (registration number: CRD42022368435).

### 2.1 Selection criteria

In this NMA, we included the RCTs that comprised the patients only with recurrent CDIs who were treated FMT via any delivery modality, antibiotic therapy, novel microbiota-based therapeutics, and monoclonal antibody. We excluded the case reports, observational studies, abstract RCTs, reviews, editorials, commentaries, conference abstracts, protocols, and practice guidelines. The RCTs that did not evaluate clinical resolution or cure of rCDI symptoms as an outcome were also excluded.

### 2.2 Data sources and search strategy

Two investigators (HD and YWY) independently conducted a systematic search of electronic databases up to March 2024, including PubMed, Cochrane, Web of Science, Embase database, and relevant review articles and meta analyses were screened to identify eligible articles that may have been missed during the initial search. The search strategies included “*Clostridioides difficile* infections” and “Fecal microbiota transplantation.” The search was restricted to English-language publications. After removing duplicates, we carefully reviewed the full texts of the remaining articles. Finally, 17 RCT articles were included in our network meta-analyses. These studies conducted comparative analyses of various interventions on rCDI and acquired available data concerning the outcomes. The discrepancies were reconciled by a third independent reviewer (GQZ). The more detailed search strategy is shown in Supplementary Table S3.

### 2.3 Data abstraction

Data were independently and systematically abstracted to a prespecified data extraction form by two reviewers (HD and YWY). Collected data from each study included authors, published year, study design, the definition of CDI, total numbers, interventions, comparators, the definition of a cure, and follow-up time. Any controversies were reassessed by consensus or arbitrated by a third reviewer (YXC), referring to the original article, until disagreement was resolved.

### 2.4 Risk of bias

Two reviewers (HD and YWY) assessed the selected articles’ methodological quality using the Cochrane Collaboration risk of bias 1.0 tool (Higgins et al., 2011). The evaluated biases included selection bias (random sequence generation and allocation concealment), performance bias (blinding of participants and personnel) and detection bias (blinding of outcome assessment), attrition bias (incomplete outcome data), reporting bias (selective reporting), and other biases. Each bias was weighted with three levels (low, unclear, and high risk) (Supplementary Table S2).

### 2.5 Definitions and outcomes

We defined the patients with rCDI who have one or more rCDI after a primary episode or had two or more episodes, and CDI was defined with a positive stool test for *C. difficile* toxin (Enzymatic immunoassay or polymerase chain reaction or pseudomembranes on colonoscopy or glutamate dehydrogenase and *C. difficile* toxins A/B or by detection of glutamate dehydrogenase and *C. difficile* cytotoxin B gene). The cure was defined as the disappearance of diarrhea explained by other causes, with at least one negative stool test for *C.s difficile* toxin, improvement of clinical manifestation, and no episodes of CDI during the follow-up day after treatment. It is worth mentioning that each study has different definitions of cure. Still, we respected the definition in each study if it is not violating our definition principles. We have chosen the resolution of rCDI and the resolution of rCDI throughout the longest follow-up period as our major efficacy. Regarding the included FMT studies, except for one AFMT, all others were DFMT. To reduce the heterogeneity of different delivery modalities of donor FMT, we split patients who received DFMT into three groups: DFMT by LGI (FMT by lower gastrointestinal routes (LGI) like retention enema, sigmoidoscopy or colonoscopy), DFMT by UGI (FMT delivered by upper gastrointestinal routes (UGI) i.e., nasogastric/nasoduodenal tube, endoscopy, oral capsules), and Vancomycin + FMT (Rokkas et al., 2019).

### 2.6 Certainty of the evidence

We evaluated the certainty of the evidence from high to very low, using the Grading of Recommendations Assessment, Development, and Evaluation (GRADE) framework (Balshem et al., 2011; Guyatt G. et al., 2011a; G. H. Guyatt et al., 2011b; Guyatt et al., 2011c; Guyatt et al., 2011d; Guyatt et al., 2011e; Guyatt et al., 2011f). Evidence from RCTs begins as high-quality evidence but may be downgraded for the following reasons: study limitations, inconsistency of results, indirectness of evidence, imprecision, and reporting bias.

### 2.7 Statistical analysis

The NMA was performed with the Bayesian Markov chain Monte Carlo method. We estimated summary odds ratios (ORs) for dichotomous outcomes using pairwise and NMA. The NMA was performed by random effects with Cochran’s Q test to assess between-study heterogeneity. The transitivity assumption underlying NMA was evaluated by comparing clinical and methodological variables distribution that could act as effect modifiers across intervention comparisons. In addition, we assessed inconsistency by Z-test, and local inconsistency of direct and indirect results was assessed with the node-splitting method for all comparison loops. Indirect results were derived from direct and network results by the back-calculation method (Dias et al., 2010). Meanwhile, we constructed comparison-adjusted funnel plots to examine funnel plot asymmetry to assess for publication bias or small-size trials influence the efficacy results. Rankograms for the CDI intervention display the cumulative probabilities of ranking first to eighth based on the findings of the NMA, and these are used to calculate SUCRA values. The surface under the cumulative ranking curve (SUCRA) was used to show the probability that each approach would be the best for each outcome. NMA was performed using Stata 17SE and R, version 4.2.1 (R Foundation for Statistical Computing) and R-Studio Integrated Development Environment for R (R-Studio, Inc). A *p*-value <0.05 was considered significant, except for heterogeneity, for which the respective value was 0.1.

## 3 Results

### 3.1 Search results

**FIGURE 1:**
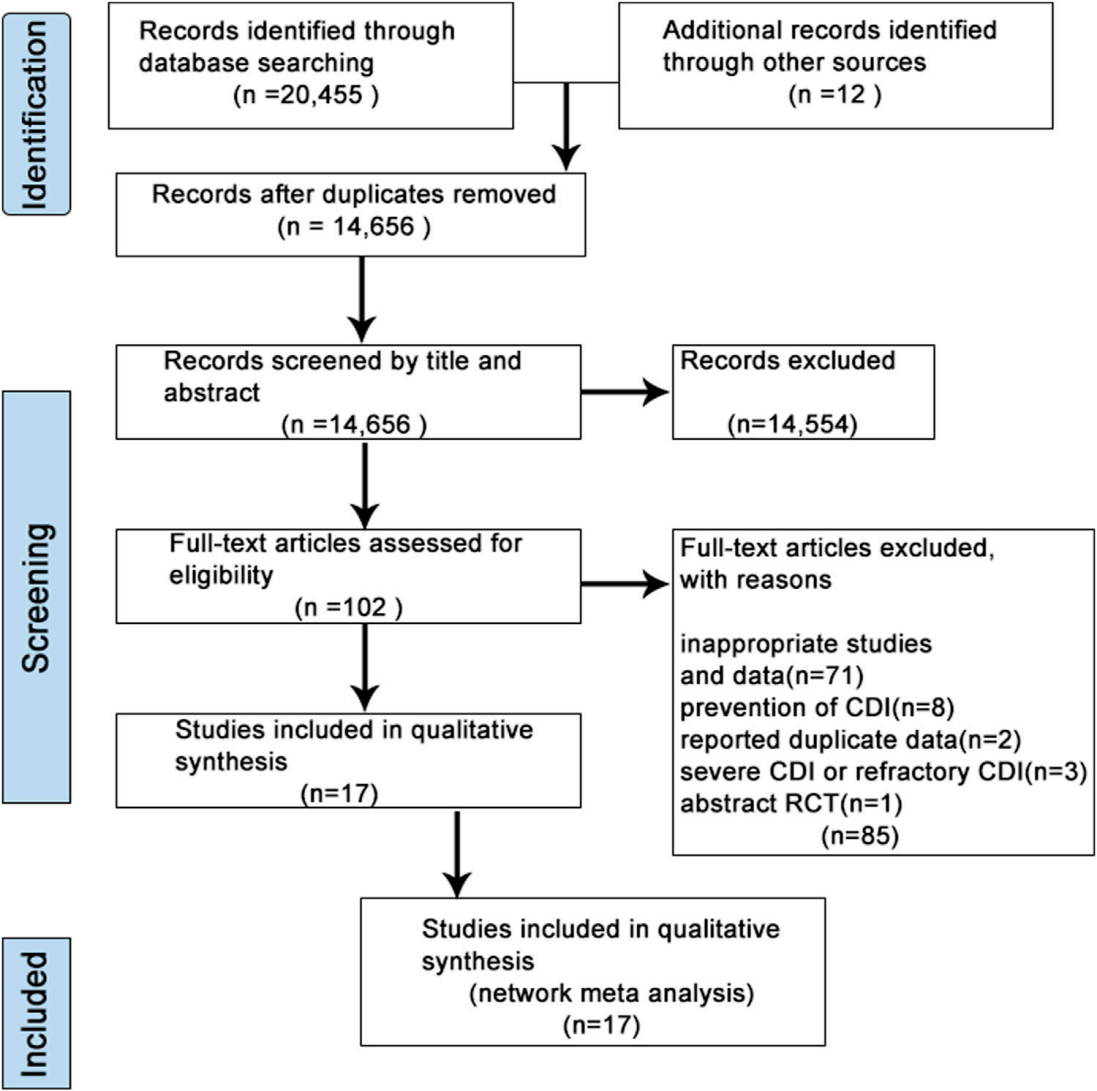
Flow diagram of the literature search and selection in the network meta-analysis.

A flow diagram for study selection was illustrated in Figure 1. Through database searching, we identified 20,467 original literature records up to 10 March 2024. 14,656 records were removed due to repeatability. After the title and abstract review, 14,554 records were excluded. Therefore, the full texts of 102 studies were assessed carefully, and studies with an inappropriate topic or insufficient data were further excluded. We finally selected 17 RCT articles (Cammarota et al., 2015; Dubberke et al., 2018; Feuerstadt et al., 2022; Hota et al., 2017; Hvas et al., 2019; Jiang et al., 2017; Jiang et al., 2018; Kao et al., 2017; Kelly et al., 2016; Khanna et al., 2022; Lee et al., 2016) for the following NMA (Supplementary Table S3).

These 17 clinical studies involved a total of 4,148 rCDI patients. Patients were recruited from the United States, Netherlands, Italy, Canada, and Denmark. The main characteristics of studies included in the NMA were shown in Table 1, which comprised study design, the definition of rCDI and cure, total numbers, detailed interventions and comparators, and the follow-up time. In our NMA, the 15 RCTs included ten interventions with 45 possible pairwise comparisons, of which 12 were pairwise comparisons with direct data. There were thirteen RCTs with two arms and two with multi-arms (Hvas et al., 2019; van Nood et al., 2013) (Table 2a).

**TABLE 1:** Main characteristics of studies included in the network meta-analysis.

Author-year	Design	Definition of recurrent *C. difficile* infection	Total, n	Intervention	Comparator	Definition of cure	Follow-up
Van Nood-2013	Open-label, randomized, controlled trial	Diarrhea (≥3 loose or watery stools per day for at least 2 consecutive days or ≥8 loose stools in 48 h) and a positive stool test for *C. difficile* toxin	42	Vancomycin (500 mg qid, 4 days), followed by bowel lavage with 4 L Macrogol solution (Klean-Prep) on final day of antibiotic treatment and subsequent infusion of FMT solution through a nasoduodenal tube the next day (n = 16)	Vancomycin (500 mg qid, 14 days) (n = 13), or vancomycin with bowel lavage on day 4 or 5 (n = 13)	Absence of diarrhea or persistent diarrhea explained by other causes, with three consecutive negative stool tests for *C. difficile* toxin	10 weeks
G.Cammarota-2015	Open-label, randomized clinical trial	Diarrhea (≥3 loose or watery stools per day for 2 or more consecutive days, or ≥8 loose stools in 48 h) and positivity in the *C. difficile* toxin stool test within 10 weeks from the end of the previous antibiotic treatment	39	Vancomycin (125 mg by mouth four times a day for 3 days), followed by bowel cleaning (n = 20)	Vancomycin (125 mg qid, 10 days), followed by 125–500 mg/day every two to 3 days for at least 3 weeks (n = 19)	Disappearance of diarrhea, or persistent diarrhea explicable by other causes, with two negative stool tests for *C. difficile* toxin	10 weeks
Kelly-2016	Randomized, controlled, double-blind clinical trial	≥3 unformed stools over 24 h for 2 consecutive days and either a positive stool test result for *C difficile* or pseudomembranes on colonoscopy	46	300 mL fecal suspension infused into terminal ileum or cecum (n = 22)	Autologous FMT: 300 mL fecal suspension from own stool infused into terminal ileum or cecum (n = 24)	<3 unformed stools per day) for 8 weeks without requirement for further antibiotics	8 weeks
Hota et al. (2017)	In a phase 2/3, single-center, open-label trial, Randomized controlled Trial	Diarrhea (6 watery stools during the previous 36 h, 3 unformed stools in a 24-h period over 2 days, or 8 unformed stools during a 48-h period), and positivity in the *C. difficile* toxin stool test (Enzymatic immunoassay or polymerase chain reaction)	30	Oral vancomycin (125 mg qid, 14 days) followed by a single 500 mL FMT (n = 16)	Oral tapering vancomycin for 6 weeks: 125 mg, qid, 14 days; then twice daily, once daily, every second day, every third day; each 1-week duration (n = 14)	No CDI recurrence within 120 days (not laboratory confirmed)	17 weeks
Dubberke- 2018	A randomized, double-blind, placebo-controlled Trial	A diagnosis of rCDI and either two or more documented recurrences of CDI after a primary episode, or two or more documented episodes of severe CDI resulting in hospitalization (CDI was defined as the presence of diarrhea (three or more unformed stools in 24 h for at least two consecutive days) and a positive stool test for *C. difficile* or its toxin)	127	RBX2660 2does or RBX2660 1 does, followed by 1 dose of placebo (n = 83)	2 doses of placebo (n = 44)	The absence of diarrhea and no retreatment for CDI any time after the first dose until 8 weeks after the second dose of assigned study treatment	8 weeks
Hvas-2019	An active-comparator, open-label, single-center randomized trial	≥3 liquid stools per day, a positive polymerase chain reaction test result for CD toxin A, toxin B, or binary toxin	64	Vancomycin (125 mg 4 times daily, 4–10 days), followed by FMT (colonoscopy or nasojejunal tube) (n = 24)	Fidaxomicin (200 mg twice daily, 10 days) (n = 24), or Vancomycin (125 mg 4 times daily, 10 days) (n = 16)	Clinical resolution and a negative result from a polymerase chain reaction test for *Clostridium difficile* toxin 8 weeks	8 weeks
Jiang-2018	A randomized, single-center trial	≥3 total episodes of CDI (≥3 watery stools per 24 h for at least two consecutive days, with a positive test for fecal *C. difficile* toxins with receipt of anti-CDI antibiotics)	65	Frozen FMT, enema, and retain for 60 min (500 mL containing 100 g of fecal microbiota) (n = 34)	Lyophilized FMT, oral, containing 100 g of fecal microbiota (n = 31)	No episodes of CDI during the 60 days after FMT treatment	60 days
Kao-2017	Noninferiority, unblinded, randomized trial	≥3 episodes of CDI. each episode was defined as a recurrence of diarrhea (>3 unformed bowel movements every 24 h) within 8 weeks of completing a prior course of treatment, and a positive *C difficile* toxin by glutamate dehydrogenase and *C difficile* toxins A/B or by detection of glutamate dehydrogenase and *C difficile* cytotoxin B gene	105	FMT, colonoscopic administration, 360 mL of fecal slurry (n = 52)	FMT, oral, 40 capsules (n = 53)	Without rCDI 12 weeks after FMT	12 weeks
Feuerstadt-2022	A double-blind, randomized, multicenter, placebo-controlled trial	Three or more episodes of *C. difficile* infection within 12 months (three or more unformed bowel movements over 2 consecutive days), a positive *C. difficile* toxin test, and resolution of symptoms while receiving 10–21 days of standard-of-care antibiotic therapy	182	SER-109 1 capsule a day, 3 days (n = 89)	Placebo 1 capsule a day, 3 days (n = 93)	CDI no recurrence within 8 weeks (not laboratory confirmed)	8 weeks
Youngster-2014	An open-label, randomized, controlled trial	≥3 episodes of mild-to-moderate CDI and failure of a 6- to 8-week taper with vancomycin with or without an alternative antibiotic, OR at least 2 episodes of severe CDI resulting in hospitalization and associated with significant morbidity (Active CDI was defined as diarrhea (>3 loose stools per day) with a positive stool test for *C. difficile* toxin)	20	FMT, colonoscopic administration, 90 cc (n = 10)	FMT, nasogastric tube administration, 90 cc (n = 10)	Clinical resolution of diarrhea off antibiotics for *C. difficile* (<3 bowel movements per 24 h), without relapse within 8 weeks	8 weeks
Khanna-2022	A randomized, double-blind, placebo-controlled trial	One or more rCDI after a primary episode or had two or more episodes of severe CDI resulting in hospitalization Participants’ CDI diarrhea had to be controlled (<3 unformed/loose stools/day for 2 consecutive days), and a positive stool test for the presence of toxigenic *C. difficile* within 30 days before enrollment	267	RBX2660: single-dose (n = 180)	Placebo: single-dose (n = 87)	Absence of CDI diarrhea within 8 weeks	8 weeks
Wilcox-2017 MODIFY I	double-blind, randomized, placebo-controlled	diarrhea (≥3 unformed bowel movements [types 5 to 7 on the Bristol stool scale18] in 24 h) with a stool test result that was positive for toxigenic *C. difficile*	1,396	actoxumab plus bezlotoxumab (10 mg per kilogram each) (n = 383)	bezlotoxumab (10 mg per kilogram of body weight) (n = 386), or actoxumab (n = 232), or placebo (n = 395)	recurrent of *C. difficile* infection	12 weeks
Wilcox-2017 MODIFY II	double-blind, randomized, placebo-controlled	diarrhea (≥3 unformed bowel movements [types 5 to 7 on the Bristol stool scale18] in 24 h) with a stool test result that was positive for toxigenic *C. difficile*	1,163	actoxumab plus bezlotoxumab (10 mg per kilogram each) (n = 768)	bezlotoxumab (10 mg per kilogram of body weight) (n = 378), or placebo (n = 395)	recurrent of *C. difficile* infection	12 weeks
Sims-2023	Open-Label, Single-Arm Trial	(1) 3 or more unformed stools per day for 2 consecutive days, (2) any positive result of a *C difficile* stool test for toxin production (i.e., EIA for toxin or cell cytotoxicity neutralization assay) or a polymerase chain reaction (PCR) assay for detection of a toxin gene from a local or central laboratory, and (3) a response to CDI antibiotic treatment, defined as 10–42 days of vancomycin, 125 mg 4 times daily, or 10–25 days of fidaxomicin, 200 mg twice daily, including prolonged tapered antibiotic regimens	263	SER-109 (with a target of 3 × 10^7^ spore colony–forming units per dose in 4 capsules, 3 days) (n = 238)	Placebo (n = 25)	CDI recurrence as determined by toxin assay up to week 4, 8, 12, and 24 after initiation of treatment	8 weeks (4, 8, 12, and 24
McGovern-2020	A multicenter, randomized, double-blind	≥3 CDI episodes within 9 months. The episode definition includes ≥3 stools/day for 2 or more consecutive days, a positive *C. difficile* stool test by either polymerase chain reaction (PCR) or toxin testing (by enzyme immunoassay), and clinical response to standard-of-care antibiotics	89	SER-109, 4 capsules (n = 59)	Placebo, 4 capsules (n = 30)	Without 3 or more unformed stools per day for 2 consecutive days, with a positive *C. difficile* stool test, in 8 weeks	8 weeks
Lee-2016	Randomized, double-blind, noninferiority trial	a history of recurrent or refractory CDI were enrolled in the study. CDI was defined by a positive result for *C difficile* toxins by enzyme immunoassay or by polymerase chain reaction targeting the *C difficile* toxin B gene (tcdB) and 3 or more unformed stools within 24 h, for a minimum of 48 h	232	frozen FMT (n = 91)	fresh FMT (n = 87)	recurrence of CDI-related diarrhea at 13 weeks after receiving up to 2 FMTs	13 weeks
Jiang-2017	Randomized, double-blind clinical trial	a history of ≥3 separate bouts of CDI in the past 12 months	72	Fresh FMT, colonoscopic administration, 50 g (n = 25)	Frozen FMT, colonoscopic administration, 50 g (n = 24) Or lyophilised FMT, colonoscopic administration, 50 g (n = 23)	freedom from bouts of *C. difficile* infection during the 5 months after FMT	5 months

**TABLE 2:** Network characteristics (a) and direct pair-wise comparisons (b) Direct pairwise comparisons.

(a) Network characteristics	
Number of interventions	10
Number of studies	15
Total number of patients in network	4,148
Total possible pairwise comparisons	45
Number of pairwise comparisons with direct data	12
Number of two-arm studies	13
Number of multiarm studies	2

(b) Direct pairwise comparisons						
	Studies (no)	OR	95% CI	Test for Overall effect (Z vaule)	*P* value	I2 (%)
A VS B	3	1.53	[0.57, 4.13]	0.84	0.40	0
A VS C	1	6.00	[1.13, 31.94]	2.10	0.04	NA
A VS E	1	25.20	[4.24, 149.79]	3.55	0.0004	NA
B VS E	1	14.44	[2.39, 87.40]	2.91	0.004	NA
B VS F	1	9.75	[1.74, 54.52]	2.59	0.010	NA
D VS E	2	5.06	[1.78, 14.43]	3.04	0.002	0
D VS G	1	4.86	[1.43, 16.50]	2.53	0.001	NA
E VS F	1	0.68	[0.12, 3.87]	0.44	0.66	NA
E VS G	1	0.91	[0.23, 3.53]	0.14	0.89	NA
F VS H	2	0.38	[0.23, 0.61]	3.93	0.0001	56
F VS I	3	0.79	[0.50, 1.26]	0.97	0.33	84
F VS J	2	0.68	[0.56, 0.83]	3.78	0.0002	33

AFMT: autologous fecal microbiota transplantation; BL: bowel lavage; CI: confidence interval; NA: not applicable; OR: odds ratio; LGI: lower gastrointestinal routes; UGI: upper gastrointestinal routes; A: FMT, by LGI; B: FMT, by UGI; C: AFMT; D: Vancomycin + FMT; E: vancomycin; F: placebo; G: fidaxomicin; H: SER109; I: RBX2660; J: monoclonal antibody.

Among the 17 RCTs, three were double-blinded, and fourteen were open-label. All the open-label trials had an unclear to high risk of bias due to the lack of blinding and a control group. We followed the previously described methodology in our assessment of attrition and reporting biases (Duo et al., 2022). Quality assessment of each included study was performed using the risk of bias 1.0 tool (Higgins et al., 2011). Each bias was weighted with three levels (low, unclear, and high risk). The risk-of-bias items for each study’s detailed information are illustrated in Supplementary Tables S2, S3. The summary graph showing the proportion of each type of bias is in Supplementary Figures S1, S2. Results showed that around 35%–50% of studies had a low risk of all types of biases, which were acceptable for the following analysis. The high risk of bias was predominantly the performance bias, which was exclusively found in the open-label trials. Heterogeneity, transitivity, and inconsistency of the network meta-analysis were also evaluated. There was no significant direct heterogeneity across head-to-head interventions, except Rebyota versus placebo had high heterogeneity due to the difference of placebo (Table 2b). Even though patients recruited for RCTs were thoughtfully selected, we excluded inflammatory bowel disease, ulcerative colitis, irritable bowel syndrome, and Crohn’s disease. It was intended as a methodological strength to assure transitivity in the network. We assessed inconsistency (statistical evidence of the violation of the transitivity assumption) by fitting both an inconsistency model and a consistency model, which can best be evaluated by node-split modeling.

### 3.2 Network meta-analysis

**FIGURE 2:**
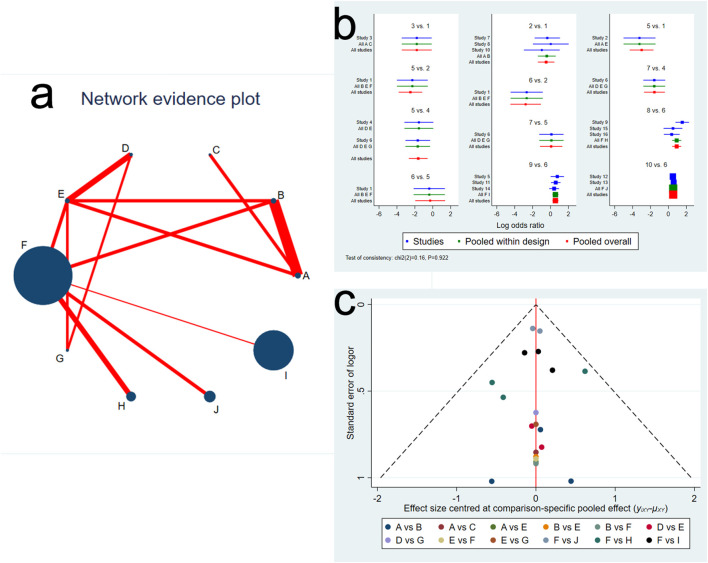
**(A)** Network evidence plot of *Clostridioides difficile* infection interventions included in the randomized controlled trials. The node size reflects the number of patients allocated to each treatment, whereas the thickness of the edges in the network plot was determined by summing up the standard errors of the estimated coefficients associated with each edge, including every pair of treatments (direct comparisons). **(B)** Network forest plot. There was no significant inconsistency (*P* = 0.922) **(C)** Funnel plot. Treatment labels: A: FMT by LGI; B: FMT by UGI; C: AFMT; D: Vancomycin +FMT; E: Vancomycin; F: Placebo; G: fidaxomicin; H: Vowst (SER109); I: Rebyota (RBX2660); J: Monoclonal antibody.

We constructed the network evidence plot for the ten therapeutic interventions of the thirteen studies (Figure 2A). Each intervention was represented by letters, i.e., A = FMT by LGI, B = FMT by UGI, C = AFMT, D = Vancomycin + FMT, E = Vancomycin, F = Placebo, G = Fidaxomicin, H = Vowst, I = Rebyota, J = Monoclonal antibody. The node size reflected the number of patients allocated to each treatment, whereas the thickness of the edges in the network plot was determined by summing up the standard errors of the estimated coefficients associated with each edge, indicating that greater uncertainty or variability in FMT by LGI versus FMT by UGI. The results showed that most patients received a placebo treatment, followed by Rebyota, monoclonal antibody, and Vowst. The direct comparison between FMT by LGI versus FMT by UGI represented the highest proportion of selected studies, followed by the comparison between Rebyota versus placebo.

We next evaluated the inconsistency and discordance between direct and indirect comparisons. We observed that the overall inconsistency results were insignificant (*P* = 0.923, indicating that the comparative effect sizes were consistent in the network forest (Figure 2B). In addition, there was no significant difference among selected studies, pooled studies with the same comparison, and pooled all studies. We also assessed the comparison-specific pooled effect visualized by funnel plot. Results showed no strong evidence of publication bias, and a symmetrical inverted funnel-shaped graph was shown in Figure 2C.

**FIGURE 3:**
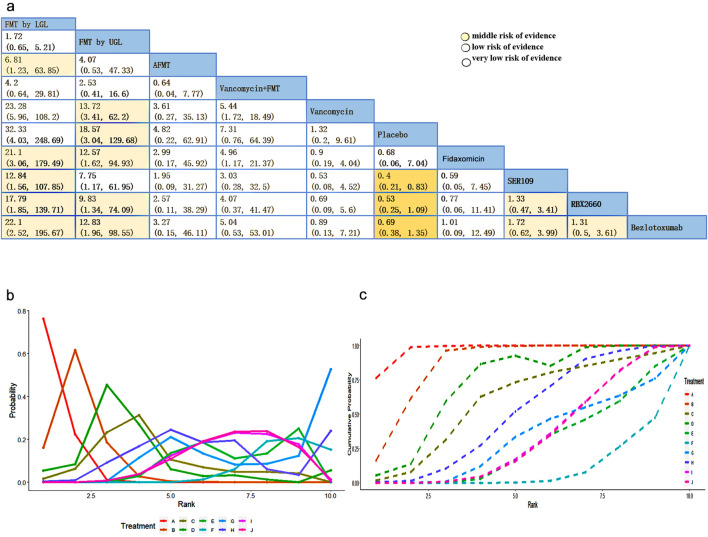
**(A)** League table showing the comparative efficacies of CDI interventions. **(B)** Rankograms for the *Clostridioides difficile* intervention network show each intervention’s cumulative rank order. **(C)** SUCRA: surface under cumulative ranking. Values for the eight therapeutic interventions. Low level of evidence: Our confidence in the effect estimate is limited: The true effect may be substantially different from the estimate of the effect. Very low level of evidence: We have very little confidence in the effect estimate: The true effect is likely to be substantially different from the estimate of effect.

We next evaluated the contribution proportion of each direct and indirect comparison in the network. The contribution plot showed that the comparison of Vancomycin + FMT versus Vancomycin had the largest contribution to the entire network (12.3%), followed by FMT by LGI versus FMT by UGI (11.6%) (Supplementary Figure S3). Next, we performed a total of 45 pairwise comparisons and predicted intervals of each treatment comparison. In the forest plot of prediction interval, the black line showed the 95% confidence intervals of fixed model, and the red line showed 95% confidence intervals of random model. The result of the intersection with the intermediate invalid line was invalid. Supplementary Figure S4 showed that most intervention comparisons had consistent results between the random and fixed effects model, with lower heterogeneity. The inconsistency analysis for all comparison loops was performed by the node-splitting method, and there was no statistical significance, suggesting no loops of evidence with inconsistency (Supplementary Figure S5). Global heterogeneity showed the pair-wise and network analysis heterogeneity variance parameter I^2^, indicating the results showed a low heterogeneity except Vowst versus placebo (*I* ^*2*^ = 59.5%) through mtc.anohe command of the gemtc package (Supplementary Figure S6). Figure 3A shows a league table for the network estimates of efficacy in all treatment comparisons. Results showed that FMT by LGI had the highest efficacy compared with placebo (odds ratios (ORs) (95% CI), 32.33 (4.03, 248.69), followed by compared with vancomycin (ORs) (95% CI), 23.28 (5.96, 108.2), and with monoclonal antibody (ORs) (95% CI), 22.1 (2.52, 195.67), respectively. However, the efficacy of comparison with FMT by LGI and FMT by UGI was not statistically significant (ORs) (95% CI), 1.72 (0.65, 5.21). Additionally, there appears to be minimal disparity in therapeutic efficacy among fresh FMT, frozen FMT, and cryopreserved FMT(Supplementary Figure S7). The rankogram and surface under cumulative ranking (SUCRA) analyses illustrated the probability of each ranking (rank numbers from the best to the worst rank) for each intervention. They showed that the efficacy of FMT by LGI and FMT by UGI, in comparison with other interventions, is the most effective option for treating rCDI. The rank of the other interventions followed by vancomycin + FMT, Vowst, fidaxomicin, AFMT, Rebyota, monoclonal antibody, vancomycin, and placebo (Figures 3B, C).

### 3.3 Quality of evidence

Based on the GRADE, we assessed the quality of evidence shown in Supplementary Table S4. Most of them were downgraded because eight open-labeled clinical studies were unblinded. All studies were downgraded due to a lack of information about effect modifiers. Some were downgraded because their 95%Crl of the OR contained the clinical cutoff threshold (Supplementary Table S4). Most pairwise comparisons are in a low or very low level of evidence, so we should interpret results carefully (Figure 3A).

## 4 Discussion

*Clostridioides difficile* infection is a common cause of hospital-acquired diarrhea and a major challenge for healthcare systems. The burden of CDI in the past decade can be explained in terms of morbidity, severity, mortality, and the increase in the possibility of recurrence (Lessa et al., 2015). As the most common complication of CDI, a major clinical challenge is that rCDI lacks drug-based therapy. Compared to gold-standard antibiotic therapy (The recommended therapy for the primary episode of CDI is either vancomycin or fidaxomicin), fecal microbiota transplantation (FMT) has been proven a novel, effective, and safe (mild side effects) treatment for severe and fulminant CDI patients as well as in the recurrence of CDI (Cheng et al., 2021).

According to previous studies, antibiotic therapy for CDI patients can lead to drug resistance and excessive adverse reactions. Therefore, FMT has become a promising treatment option for refractory CDI patients due to its effective cure rate and good safety (Pomares Bascuñana et al., 2021). In FMT studies, there are different routes of administration and other types of microbial agents, including nasogastric or nasojejunal tubes (NGT or NJT), upper gastrointestinal (GI) endoscopy, colonoscopy and enemas, oral capsules, and frozen fecal material. A meta-analysis demonstrated no statistically significant difference in efficacy among lyophilized FMT, fresh FMT, and colonoscopic frozen FMT. There was also low-quality evidence supporting oral capsule FMT for rCDI with low adverse effects (Du, Luo, Walsh and Grinspan, 2021). Ramai et al. showed that FMT was well tolerated in treating rCDI, and colonoscopy FMT is superior to NGT and enema FMT methods. In addition, capsule FMT is comparable to colonoscopy (Ramai et al., 2021).

Regarding the RCTs we included, the results showed that FMT was the most effective in rCDI patients. At the same time, vancomycin and placebo were the least effective in rCDI patients. Previous studies also suggest that FMT is a highly effective and durable treatment for rCDI (G. Cammarota et al., 2015; Drekonja et al., 2015; Jalanka et al., 2018; van Nood et al., 2013). It is recommended as the best treatment option for rCDI after antibiotic failure (Cammarota et al., 2017; Debast et al., 2014; König et al., 2017; Surawicz et al., 2013). Antibiotic therapy, on the other hand, is the least effective for CDI patients, not only prone to relapse of *C. difficile* after discontinuation of antibiotics but also leading to low microbial diversity (i.e., dysbiosis) due to antibiotic exposure, impairing colonization resistance, which is a major function of a healthy microbiome (Chang et al., 2008; Seekatz et al., 2015). Fidaxomicin and vancomycin relieve symptoms by killing *C. difficile* (Louie et al., 2012; Louie et al., 2011). However, antibiotics did not affect dormant *C. difficile* spores, which rapidly germinated to become toxin-producing vegetative bacteria when dysbiosis persisted after cessation of treatment. Due to less damage to the gut microbiome during infection treatment, fidaxomicin and vancomycin have comparable results in their resolution, but a more durable resolution can be achieved with fidaxomicin (Louie et al., 2011). However, severe clinical manifestations, life-threatening complications, and even death (i.e., toxic megacolon, sepsis) are always accompanied by antibiotic resistance of *C. difficile* (Olsen et al., 2015).

In the curative effect ranking of treatment in rCDI patients, our results showed that FMT by LGI was at the top of our ranking list. FMT is considered to perform healthy fecal microbiota transplantation through colonoscopy enema and nasoduodenum. There is also a large amount of evidence pointing to the effectiveness of FMT. However, the safety of FMT should still be considered, especially in elderly and frail patients who may not tolerate colonoscopy and associated sedation during the procedure (Youngster et al., 2014).

FMT by UGI ranked second in the efficacy of rCDI patients. FMT by UGI is not only a safe approach but also exhibited comparable efficacy to the traditional FMT method. Although the incidence of nausea in the oral capsule group was higher than that in the rectal infusion group, the difference was not statistically significant, and the incidence of vomiting was lower and similar to that in the rectal infusion group (Ramai et al., 2021). Colonoscopy is irreplaceable in diagnosis, but it is also invasive, resource-intensive, expensive, inconvenient, and intolerable to patients. On the contrary, FMT by UGI has good safety, fewer adverse reactions, and is easy to administer. Therefore, after combining more research and economic evaluation, the value of FMT by UGI can be determined and may be used as a mainstream microbial treatment in the future. These findings highlight the promising potential of FMT by UGI as an effective and well-tolerated treatment option for rCDI patients. Although FMT has been studied for many years and has proven to be an effective treatment for rCDI, significant gaps remain in the standardization and regulation of its manufacturing processes. The lack of FDA-compliant manufacturing methods has limited the broader clinical application of FMT. While the FDA mandates rigorous pathogen screening at both the donor and product levels, the specific number of pathogens screened may vary according to different guidelines. Despite these stringent screening procedures, there have been reports of severe infections caused by the use of unregulated FMT, underscoring the ongoing challenges in ensuring the safety and regulatory compliance of FMT. Literature has documented four cases of serious infections resulting from the use of unregulated FMT, highlighting the critical need for enhanced oversight and standardized production to ensure the safety and efficacy of these treatments (Administration, 2022).

It is worth noting that Vowst, an investigational oral microbiota-based therapeutic consisting of live purified bacterial spores from healthy donors of the Firmicutes phylum, ranks in terms of efficacy only slightly less than FMT for rCDI patients. The administration of Vowst typically occurs within 24–72 h after the completion of a standard antibiotic course to ensure optimal effectiveness in restoring the gut microbiome. It needs to be taken on an empty stomach, with bowel preparation beforehand to ensure that the spore-based formulation reaches the colon intact. These capsules contain bacterial spores that can resist stomach acid, ensuring they remain viable until they reach the intestine. Once in the intestine, these spores help rebuild a healthy microbiome by competing for nutrients and space, and they may also modulate the bile acid composition in the gut to inhibit further germination of *C.difficile* spores. Firmicutes and Bacteroidetes are the two dominant phyla in the gastrointestinal microbiota, while pro-inflammatory proteobacteria account for a limited proportion of the healthy microbiota (Eckburg et al., 2005). Depletion of Firmicutes and their metabolites promoted the recurrence of CDI. Primary and secondary bile acids (BAs) play an essential role in the life cycle of *C. difficile*. Primary BA synthesized in the liver is secreted into the intestine and converted into secondary BA by commensal microorganisms. Primary BA promotes *Clostridioides difficile* germination of spores, whose vegetative growth is inhibited by certain secondary Bas (Sorg and Sonenshein, 2009; Weingarden et al., 2016). The concentration imbalance between primary BA and secondary BA leads to an increase in relative concentration, which provides favorable conditions for spore germination, bacterial replication, and toxin production (Theriot and Young, 2015). Therefore, the supplementation of firmicutes is necessary, and the restoration of microbial diversity through microbial therapy can ensure the balance of BA and the germination and vegetative growth of spores, which also makes up for the fact that antibiotics can only kill *C. difficile* but are ineffective against spores’ shortcomings. Simultaneously, these spore-forming bacteria metabolically compete with *C. difficile* for essential nutrients, modulating bile-acid profiles to reestablish resistance to colonization. Therefore, Vowst, as a DFMT by UGI, significantly affects patients with *C. difficile* infection. CDI recurrence usually occurs within 1–3 weeks of antibiotic discontinuation, the window of vulnerability (Abujamel et al., 2013; Kelly, 2012). Vowst accelerates microbiome repair during the window of vulnerability, thereby limiting *C. difficile* spore germination and clinical relapse. This observed safety profile of Vowst might be predictable since spore-forming Firmicutes bacteria are abundant in healthy microbiomes (Lopetuso et al., 2013). In addition, the Vowst manufacturing process of Vowst provides an effective microbial composition while mitigating the risk of transmitting undetected or emerging pathogens beyond pure donor screening. Rebyota also is a live biotherapeutic product derived from a diverse consortium of microbes extracted from human stool that is currently being investigated for its potential in reducing rCDI, and it demonstrated the superiority of Rebyota compared with a placebo. Rebyota is administered within 24–72 h after the completion of a standard antibiotic course to ensure optimal effectiveness in restoring the gut microbiome. Delivered as a single enema dose, Rebyota directly introduces a broad consortium of healthy donor microbiota into the colon. This method rapidly alters the local microbial environment, suppressing the overgrowth of *Clostridioides difficile* and promoting quick colonization to potentially displace harmful pathogens. Adverse events associated with Rebyota were similar to those observed in FMT studies, mainly mild-to-moderate gastrointestinal disorders, with abdominal pain and diarrhea being the most common, Rebyota had a low rate of AEs leading to discontinuation of participation, and no new or unexpected adverse events, pathogen transmission from the donor to the recipient, or product- or procedure-related serious adverse events were reported (E. R. Dubberke et al., 2018; Orenstein et al., 2016; Orenstein et al., 2022). A recognized safety concern with FMT use is the potential transmission of infectious diseases (DeFilipp et al., 2019). Therefore, Vowst and Rebyota, as novel microbiota-based therapeutic products, hold promise and potential in the treatment of rCDI patients while also reducing some of the risks associated with the treatment process.

Fidaxomicin ranks fifth in the ranking, which may be related to less original research data, but it also shows a significant effect. While AFMT ranks sixth, it may be due to the continued evolution of microbial community structure after autologous FMT in the absence of CDI recurrence or possibly due to the discontinuation of antibiotics. In addition, the significance of the AFMT trend needs to be clarified as the sample size is too tiny whereas resolution after AFMT differed by the site (9 of 10 vs. 6 of 14 [*P* = 0.033]) (Kelly et al., 2016). The FDA approved Bezlotoxumab in October 2016, named “Zinplava.” Bezlotoxumab is also an important treatment option for preventing rCDI. Actoxumab and bezlotoxumab are fully human monoclonal antibodies that bind and neutralize *Clostridioides difficile* toxins A and B, respectively. Although monoclonal antibodies (including actoxumab, bezlotoxumab, and bezlotoxumab + actoxumab) ranked lower in our research, the overall efficacy of monoclonal antibodies was found to be superior to vancomycin and placebo in treating rCDI.

Evolution of Guideline-Based antimicrobial recommendations to treat CDI. Treatment recommendations were basic in the first and second wave of guidelines from 1995 through 2014. They focused on vancomycin and metronidazole to add considerations for severity and a vancomycin taper which still reflected the limited treatment options available due to a lack of data (Bauer et al., 2009; Fekety, 1997; Gerding et al., 1995; Zar et al., 2007). Up to now, with recent 2020, fidaxomicin as an initial treatment option before vancomycin in both initial and recurrent diseases. According to the latest guidelines from ESCMID (late 2021), vancomycin (alone) is no longer the first-line treatment but rather a second-line therapy. FMT has been reported as the best treatment in multiple recurrences (McDonald et al., 2018). In addition, new therapeutic strategies are emerging. Still, more evidence and data are needed to update and supplement the guidelines in more detail, including stratified treatment selection in patients traditionally according to the infection to clinical severity and number of episodes of infection (Merino and Salavert, 2022).

### 4.1 Strength and limitations

The strength of our study is that we conducted a detailed network meta-analysis of different interventions and various FMT modalities in rCDI patients. Our analysis solved excessive heterogeneity in the previous study (Rokkas et al., 2019) due to FMT, including nasogastric or nasojejunal tubes, upper gastrointestinal (GI) endoscopy, colonoscopy, and enema. Additionally, our research highlighted the potential of Vowst and Rebyota as microbiome-based therapeutics in the future treatment of rCDI, offering more convenient administration or improved safety profiles, among other benefits. However, our study does have some limitations. The diverse designs of each experiment require cautious interpretation of the results, despite no significant heterogeneity. While FMT demonstrates good efficacy, the lack of FDA-compliant manufacturing methods and adequate safety screening for the product remains a significant concern, especially with regard to the risk of severe infectious complications. Furthermore, we need more detailed and multiple head-to-head clinical trials further to analyze the efficacy and safety of different interventions. Due to the variation in study designs and the overall low quality of evidence, we were unable to conduct a comprehensive quantitative analysis of adverse reactions associated with different interventions. Therefore, more well-designed, and high-quality clinical trials are needed to support our conclusions fully. The different interventions were evaluated according to more head-to-head clinical protocols, treatment outcomes, and adverse events over a more extended follow-up period. It is worth noting that the expertise of gastroenterologists, availability of treatment, and patient preference is also an important factor.

## 5 Conclusion

The outcomes of our network meta-analysis serve to elucidate the conspicuous therapeutic prowess exhibited by FMT in the management of rCDI, irrespective of its administration route - be it the lower gastrointestinal path or the upper gastrointestinal trajectory. Notably, the UGI route presents itself as a prospective supplementary conduit for FMT, distinguished by heightened accessibility and commendable safety attributes. Although FMT has shown significant efficacy in treating rCDI, the risks associated with its use, particularly those arising from unregulated production and lack of standardized oversight, remain a serious concern. Additionally, microbiota-based therapeutics similarly exhibit remarkable prowess in the domain of rCDI amelioration. However, constrained by the prevailing dearth of germane inquiry, the augmentation of clinical practices assumes pivotal relevance to furnish comprehensive underpinning for judicious determinations. By comparison, the therapeutic efficacy of antibiotics in the realm of rCDI appears somewhat subdued, conceivably due to their propensity to disrupt the harmonious symbiosis of autochthonous intestinal microflora, consequently enfeebling the curative effect.

## Data Availability

The original contributions presented in the study are included in the article/Supplementary Material, further inquiries can be directed to the corresponding authors.
