# Supplementary material for: Comparative effectiveness of treatments for recurrent Clostridioides difficile infection: a network meta-analysis of randomized controlled trials
Source: Front Pharmacol. 2024 Oct 17;15:1430724. doi: 10.3389/fphar.2024.1430724 (PMC11525118; doi:10.3389/fphar.2024.1430724)
Supplement: Supplementary file 1 [file DataSheet1.docx]

**Supplementary Online Content**

**Supplementary Table 1.** **PRISMA - NMA 2020 checklist**

**Supplementary Table 2. Quality assessment of included studies by Risk of bias 1.0**

**Supplementary Table 3. Search strategies**

**Supplementary Table 4. Certainty of the evidence**

**Supplementary Figure 1. Bias of graph**

**Supplementary Figure 2. Bias of summary**

**Supplementary Figure 3. Contribution plot for the comparisons network**

**Supplementary Figure 4. The forest plots of 28 pairwise comparisons and predicted intervals showed no difference in efficacy between the random-effects model and the fixed-effects model Supplementary Figure 5. The assessment of inconsistency with a node-splitting method for all comparison loops.**

**Supplementary Figure 6. The assessment of heterogeneity.**

**Supplementary Figure 7. Forest plot**

**Supplementary Table 1. PRISMA - NMA 2020 checklist**

**PRISMA NMA Checklist of Items to Include When Reporting A Systematic Review Involving a Network Meta-analysis**

**PRISMA - NMA 2020 checklist**

**PRISMA NMA Checklist of Items to Include When Reporting A Systematic Review Involving a Network Meta-analysis**

| **Section/Topic** | **Item #** | **Checklist Item** | **Reported on Page #** |
| --- | --- | --- | --- |
| **TITLE** |  |  |  |
| Title | 1 | Identify the report as a systematic review *incorporating a network meta-analysis (or related form of meta-analysis).* | ***Title*** |
|  |  |  |  |
| **ABSTRACT** |  |  |  |
| Structured summary | 2 | Provide a structured summary including, as applicable:  **Background:** main objectives  **Methods:** data sources; study eligibility criteria, participants, and interventions; study appraisal; and *synthesis methods, such as network meta-analysis.*  **Results:** number of studies and participants identified; summary estimates with corresponding confidence/credible intervals; *treatment rankings may also be discussed. Authors may choose to summarize pairwise comparisons against a chosen treatment included in their analyses for brevity.*  **Discussion/Conclusions:** limitations; conclusions and implications of findings.  **Other:** primary source of funding; systematic review registration number with registry name. | **ABSTRACT** |
|  |  |  |  |
| **INTRODUCTION** |  |  |  |
| Rationale | 3 | Describe the rationale for the review in the context of what is already known*, including mention of why a network meta-analysis has been conducted.* | **Introduction** |
| Objectives | 4 | Provide an explicit statement of questions being addressed, with reference to participants, interventions, comparisons, outcomes, and study design (PICOS). | **Introduction** |
|  |  |  |  |
| **METHODS** |  |  |  |
| Protocol and registration | 5 | Indicate whether a review protocol exists and if and where it can be accessed (e.g., Web address); and, if available, provide registration information, including registration number. | **Methods** |
| Eligibility criteria | 6 | Specify study characteristics (e.g., PICOS, length of follow-up) and report characteristics (e.g., years considered, language, publication status) used as criteria for eligibility, giving rationale. *Clearly describe eligible treatments included in the treatment network, and note whether any have been clustered or merged into the same node (with justification).* | **Selection Criteria** |
| Information sources | 7 | Describe all information sources (e.g., databases with dates of coverage, contact with study authors to identify additional studies) in the search and date last searched. | **Data Sources and Search Strategy** |
| Search | 8 | Present full electronic search strategy for at least one database, including any limits used, such that it could be repeated. | **Data Sources and Search Strategy** |
| Study selection | 9 | State the process for selecting studies (i.e., screening, eligibility, included in systematic review, and, if applicable, included in the meta-analysis). | **Data Sources and Search Strategy** |
| Data collection process | 10 | Describe method of data extraction from reports (e.g., piloted forms, independently, in duplicate) and any processes for obtaining and confirming data from investigators. | **Data Extraction** |
| Data items | 11 | List and define all variables for which data were sought (e.g., PICOS, funding sources) and any assumptions and simplifications made. | **Methods** |
| **Geometry of the network** | **S1** | Describe methods used to explore the geometry of the treatment network under study and potential biases related to it. This should include how the evidence base has been graphically summarized for presentation, and what characteristics were compiled and used to describe the evidence base to readers. | **Fig 2a** |
| Risk of bias within individual studies | 12 | Describe methods used for assessing risk of bias of individual studies (including specification of whether this was done at the study or outcome level), and how this information is to be used in any data synthesis. | **Risk of Bias** |
| Summary measures | 13 | State the principal summary measures (e.g., risk ratio, difference in means). *Also describe the use of additional summary measures assessed, such as treatment rankings and surface under the cumulative ranking curve (SUCRA) values, as well as modified approaches used to present summary findings from meta-analyses.* | **Result** |
| Planned methods of analysis | 14 | Describe the methods of handling data and combining results of studies for each network meta-analysis. This should include, but not be limited to:   - *Handling of multi-arm trials;* - *Selection of variance structure;* - *Selection of prior distributions in Bayesian analyses; and* - *Assessment of model fit.* | **Statistical analysis** |
| **Assessment of Inconsistency** | **S2** | Describe the statistical methods used to evaluate the agreement of direct and indirect evidence in the treatment network(s) studied. Describe efforts taken to address its presence when found. | **Result** |
| Risk of bias across studies | 15 | Specify any assessment of risk of bias that may affect the cumulative evidence (e.g., publication bias, selective reporting within studies). | **Risk of Bias** |
| Additional analyses | 16 | Describe methods of additional analyses if done, indicating which were pre-specified. This may include, but not be limited to, the following:   - Sensitivity or subgroup analyses; - Meta-regression analyses; - *Alternative formulations of the treatment network; and* - *Use of alternative prior distributions for Bayesian analyses (if applicable).* | **Result** |
|  |  |  |  |
| **RESULTS†** |  |  |  |
| Study selection | 17 | Give numbers of studies screened, assessed for eligibility, and included in the review, with reasons for exclusions at each stage, ideally with a flow diagram. | **Search Results** |
| **Presentation of network structure** | **S3** | Provide a network graph of the included studies to enable visualization of the geometry of the treatment network. | **Network meta-analysis** |
| **Summary of network geometry** | **S4** | Provide a brief overview of characteristics of the treatment network. This may include commentary on the abundance of trials and randomized patients for the different interventions and pairwise comparisons in the network, gaps of evidence in the treatment network, and potential biases reflected by the network structure. | **Network meta-analysis** |
| Study characteristics | 18 | For each study, present characteristics for which data were extracted (e.g., study size, PICOS, follow-up period) and provide the citations. | **Results**  **Table 1** |
| Risk of bias within studies | 19 | Present data on risk of bias of each study and, if available, any outcome level assessment. | **Results** |
| Results of individual studies | 20 | For all outcomes considered (benefits or harms), present, for each study: 1) simple summary data for each intervention group, and 2) effect estimates and confidence intervals. *Modified approaches may be needed to deal with information from larger networks.* | **Results**  **Table 1** |
| Synthesis of results | 21 | Present results of each meta-analysis done, including confidence/credible intervals. *In larger networks, authors may focus on comparisons versus a particular comparator (e.g. placebo or standard care), with full findings presented in an appendix. League tables and forest plots may be considered to summarize pairwise comparisons.* If additional summary measures were explored (such as treatment rankings), these should also be presented. | **Results** |
| **Exploration for inconsistency** | **S5** | Describe results from investigations of inconsistency. This may include such information as measures of model fit to compare consistency and inconsistency models, *P* values from statistical tests, or summary of inconsistency estimates from different parts of the treatment network. | **Results** |
| Risk of bias across studies | 22 | Present results of any assessment of risk of bias across studies for the evidence base being studied. | **Results** |
| Results of additional analyses | 23 | Give results of additional analyses, if done (e.g., sensitivity or subgroup analyses, meta-regression analyses*, alternative network geometries studied, alternative choice of prior distributions for Bayesian analyses,* and so forth). | **Results** |
|  |  |  |  |
| **DISCUSSION** |  |  |  |
| Summary of evidence | 24 | Summarize the main findings, including the strength of evidence for each main outcome; consider their relevance to key groups (e.g., healthcare providers, users, and policy-makers). | **DISCUSSION** |
| Limitations | 25 | Discuss limitations at study and outcome level (e.g., risk of bias), and at review level (e.g., incomplete retrieval of identified research, reporting bias). *Comment on the validity of the assumptions, such as transitivity and consistency. Comment on any concerns regarding network geometry (e.g., avoidance of certain comparisons).* | **DISCUSSION** |
| Conclusions | 26 | Provide a general interpretation of the results in the context of other evidence, and implications for future research. | **Conclusions** |
|  |  |  |  |
| **FUNDING** |  |  |  |
| Funding | 27 | Describe sources of funding for the systematic review and other support (e.g., supply of data); role of funders for the systematic review. This should also include information regarding whether funding has been received from manufacturers of treatments in the network and/or whether some of the authors are content experts with professional conflicts of interest that could affect use of treatments in the network. | **Funding** |

PICOS = population, intervention, comparators, outcomes, study design.

* Text in italics indicateS wording specific to reporting of network meta-analyses that has been added to guidance from the PRISMA statement.

† Authors may wish to plan for use of appendices to present all relevant information in full detail for items in this section.

PICOS = population, intervention, comparators, outcomes, study design.

* Text in italics indicateS wording specific to reporting of network meta-analyses that has been added to guidance from the PRISMA statement.

† Authors may wish to plan for use of appendices to present all relevant information in full detail for items in this section.

**Supplementary Table 2. Quality assessment of included studies by Risk of bias 1.0**

| Author/year | Random sequence generation (selection bias) | Allocation concealment (selection bias) | Blinding of participants and personnel (performance bias) | Blinding of outcome assessment (detection bias) | Incomplete outcome date (attrition bias) | Selective reporting (reporting bias) | Other bias |
| --- | --- | --- | --- | --- | --- | --- | --- |
| Van Nood-2013 | **-** | **NA** | **+** | **-** | **NA** | **-** | **-** |
| G.Cammarota-2015 | **-** | **NA** | **+** | **-** | **NA** | **-** | **-** |
| Kelly-2016 | **-** | **-** | **-** | **NA** | **NA** | **-** | **-** |
| Hota-2017 | **NA** | **NA** | **+** | **NA** | **NA** | **-** | **-** |
| Dubberke-2018 | **-** | **-** | **-** | **-** | **NA** | **-** | **NA** |
| Hvas-2019 | **NA** | **NA** | **+** | **NA** | **-** | **-** | **-** |
| Jiang-2018 | **-** | **NA** | **+** | **-** | **+** | **-** | **NA** |
| Kao-2017 | **NA** | **NA** | **+** | **NA** | **-** | **-** | **NA** |
| Feuerstadt-2022 | **NA** | **-** | **-** | **NA** | **NA** | **-** | **NA** |
| Youngster-2014 | **-** | **NA** | **+** | **NA** | **-** | **-** | **-** |
| Khanna-2022 | **NA** | **NA** | **-** | **-** | **NA** | **-** | **-** |
| Wilcox-2017  MODIFY I | **NA** | **-** | **-** | **-** | **NA** | **-** | **-** |
| Wilcox-2017  MODIFY II | **NA** | **-** | **-** | **-** | **NA** | **-** | **-** |
| Sims-2023 | **+** | **NA** | **NA** | **NA** | **NA** | **NA** | **-** |
| McGovern-2020 | **NA** | **-** | **-** | **NA** | **NA** | **-** | **NA** |
| Lee-2016 | **-** | **-** | **-** | **-** | **NA** | **NA** | **-** |
| Jiang-2017 | **NA** | **-** | **-** | **-** | **NA** | **NA** | **-** |

- Low risk

NA Unclear risk

+ High risk

**Supplementary Table 3. Search strategies**

| **Search strategy** | |
| --- | --- |
| **PubMed** | For **PubMed**, the search used was:  #1 Search: "Clostridium Infections"[Mesh]  #2 Search: ((((((((((((((((((((Clostridium Infection) OR (Infection, Clostridium)) OR (Infections, Clostridium)) OR (Clostridioides Infections)) OR (Clostridioides Infection)) OR (Clostridium difficile Infections)) OR (Clostridium difficile Infection)) OR (Infection, Clostridium difficile)) OR (Clostridioides difficile Infection)) OR (Infection, Clostridioides difficile)) OR (Clostridium sordellii Infections)) OR (Clostridium sordellii Infection)) OR (Infection, Clostridium sordellii)) OR (Clostridioides sordellii Infection)) OR (Infection, Clostridioides sordellii)) OR (Clostridium perfringens Infections)) OR (Clostridium perfringens Infection)) OR (Clostridioides perfringens Infections)) OR (Clostridioides perfringens Infections)) OR (Clostridium perfringens Food Poisoning)) OR (Clostridioides perfringens Food Poisoning)  #3 Search: #2 OR #1  #4 Search: "Fecal Microbiota Transplantation"[Mesh]  #5 Search: 'Fecal Microbiota Transplantations' OR 'Microbiota Transplantation, Fecal' OR 'Microbiota Transplantations, Fecal'OR 'Transplantation, Fecal Microbiota'OR 'Transplantations, Fecal Microbiota'OR 'Fecal Microbiota Transplant'OR 'Fecal Microbiota Transplants'OR 'Microbiota Transplant, Fecal'OR 'Microbiota Transplants, Fecal'OR 'Transplant, Fecal Microbiota'OR 'Transplants, Fecal Microbiota'OR 'Fecal Microbiome Transplantation'OR 'Fecal Microbiome Transplantations'OR 'Microbiome Transplantation, Fecal'OR 'Microbiome Transplantations, Fecal'OR 'Transplantation, Fecal Microbiome'OR 'Transplantations, Fecal Microbiome'OR 'Fecal Transplant'OR 'Fecal Transplants'OR 'Transplant, Fecal'OR 'Transplants, Fecal'OR 'Donor Feces Infusion'OR 'Donor Feces Infusions'OR 'Feces Infusion, Donor'OR 'Feces Infusions, Donor'OR 'Infusion, Donor Feces'OR 'Infusions, Donor Feces'OR 'Intestinal Microbiome Transplant'OR 'Intestinal Microbiome Transplants'OR 'Microbiome Transplant, Intestinal'OR 'Microbiome Transplants, Intestinal'OR 'Transplant, Intestinal Microbiome'OR 'Transplants, Intestinal Microbiome'OR 'Intestinal Microbiota Transfer'OR 'Intestinal Microbiota Transfers'OR 'Microbiota Transfer, Intestinal'OR 'Microbiota Transfers, Intestinal'OR 'Transfer, Intestinal Microbiota'OR 'Transfers, Intestinal Microbiota'OR 'Intestinal Microbiota Transplantation'OR 'Intestinal Microbiota Transplantations'OR 'Microbiota Transplantation, Intestinal'OR 'Microbiota Transplantations, Intestinal'OR 'Transplantation, Intestinal Microbiota'OR 'Transplantations, Intestinal Microbiota'OR 'Intestinal Microbiome Transplantation'OR 'Intestinal Microbiome Transplantations'OR 'Microbiome Transplantation, Intestinal'OR 'Microbiome Transplantations, Intestinal'OR 'Transplantation, Intestinal Microbiome'OR 'Transplantations, Intestinal Microbiome'OR 'Intestinal Microbiota Transplant'OR 'Intestinal Microbiota Transplants'OR 'Microbiota Transplant, Intestinal'OR 'Microbiota Transplants, Intestinal'OR 'Transplant, Intestinal Microbiota'OR 'Transplants, Intestinal Microbiota'OR 'Intestinal Microbiome Transfer'OR 'Intestinal Microbiome Transfers'OR 'Microbiome Transfer, Intestinal'OR 'Microbiome Transfers, Intestinal'OR 'Transfer, Intestinal Microbiome'OR 'Transfers, Intestinal Microbiome'OR 'Fecal Microbiota Transfer'OR 'Fecal Microbiota Transfers'OR 'Microbiota Transfer, Fecal'OR 'Microbiota Transfers, Fecal'OR 'Transfer, Fecal Microbiota'OR 'Transfers, Fecal Microbiota'OR 'Fecal Transplantation'OR 'Fecal Transplantations'OR 'Transplantation, Fecal'OR 'Transplantations, Fecal'  #6 Search: #5 OR #4  #7 Search: "Vancomycin"[Mesh]  #8 Search: (((((((((((((((((((((Vancomycin Hydrochloride) OR (Hydrochloride, Vancomycin)) OR (Vancomycin Sulfate)) OR (Sulfate, Vancomycin)) OR (Vancomycin-ratiopharm)) OR (Vancomycin Hexal)) OR (Vancomycine Dakota)) OR (AB-Vancomycin)) OR (Vanco Azupharma)) OR (Diatracin)) OR (VANCO-cell)) OR (VANCO-cell)) OR (Vancocin)) OR (Vancocin HCl)) OR (Vancomycin Lilly)) OR (Vancocine)) OR (Vancomicina Abbott)) OR (Vancomicina Chiesi)) OR (Vancomicina Combino Phar)) OR (Vancomicina Norman)) OR (Vancomicina Norman)) OR (Vancomycin Phosphate (1:2), Decahydrate)  #9 Search: #7 OR #8  #10 Search: "Fidaxomicin"[Mesh]  #11 Search: (((((((((((Lipiarmycin A3) OR (Lipiarmycin)) OR (Tiacumicin B)) OR (PAR 101)) OR (PAR101)) OR (PAR-101)) OR (Dificid)) OR (Lipiarmycin B4)) OR (Tiacumicin C)) OR (Lipiarmycin B)) OR (Lipiarmycin A4)) OR (Lipiarmycin B3)  #12 Search: #10 OR #11  #13 #6 OR #9 OR #12  #14 #3 AND #13 |
| **EMBASE** | For **EMBASE**, the search used was:  ('clostridium infection'/exp OR 'clostridium difficile infection'/exp OR 'clostridioides difficile' OR 'clostridium difficile' OR 'clostridium infections' OR 'clostridium infection' OR 'infection, clostridium' OR 'infections, clostridium' OR 'clostridioides infections' OR 'clostridioides infection' OR 'clostridium difficile infections' OR 'clostridium difficile infection' OR 'infection, clostridium difficile' OR 'clostridioides difficile infection' OR 'infection, clostridioides difficile' OR 'clostridium sordellii infections' OR 'clostridium sordellii infection' OR 'infection, clostridium sordellii' OR 'clostridioides sordellii infection' OR 'infection, clostridioides sordellii') AND ('fecal microbiota transplantation'/exp OR 'fecal microbiota transplantation' OR 'fecal microbiota transplantations' OR 'microbiota transplantation, fecal' OR 'microbiota transplantations, fecal' OR 'transplantation, fecal microbiota' OR 'transplantations, fecal microbiota' OR 'fecal microbiota transplant' OR 'fecal microbiota transplants' OR 'microbiota transplant, fecal' OR 'microbiota transplants, fecal' OR 'transplant, fecal microbiota' OR 'transplants, fecal microbiota' OR 'fecal microbiome transplantation' OR 'fecal microbiome transplantations' OR 'microbiome transplantation, fecal' OR 'microbiome transplantations, fecal' OR 'transplantation, fecal microbiome' OR 'transplantations, fecal microbiome' OR 'fecal transplant' OR 'fecal transplants' OR 'transplant, fecal' OR 'transplants, fecal' OR 'donor feces infusion' OR 'donor feces infusions' OR 'feces infusion, donor' OR 'feces infusions, donor' OR 'infusion, donor feces' OR 'infusions, donor feces' OR 'intestinal microbiome transplant' OR 'intestinal microbiome transplants' OR 'microbiome transplant, intestinal' OR 'microbiome transplants, intestinal' OR 'transplant, intestinal microbiome' OR 'transplants, intestinal microbiome' OR 'intestinal microbiota transfer' OR 'intestinal microbiota transfers' OR 'microbiota transfer, intestinal' OR 'microbiota transfers, intestinal' OR 'transfer, intestinal microbiota' OR 'transfers, intestinal microbiota' OR 'intestinal microbiota transplantation' OR 'intestinal microbiota transplantations' OR 'microbiota transplantation, intestinal' OR 'microbiota transplantations, intestinal' OR 'transplantation, intestinal microbiota' OR 'transplantations, intestinal microbiota' OR 'intestinal microbiome transplantation' OR 'intestinal microbiome transplantations' OR 'microbiome transplantation, intestinal' OR 'microbiome transplantations, intestinal' OR 'transplantation, intestinal microbiome' OR 'transplantations, intestinal microbiome' OR 'intestinal microbiota transplant' OR 'intestinal microbiota transplants' OR 'microbiota transplant, intestinal' OR 'microbiota transplants, intestinal' OR 'transplant, intestinal microbiota' OR 'transplants, intestinal microbiota' OR 'intestinal microbiome transfer' OR 'intestinal microbiome transfers' OR 'microbiome transfer, intestinal' OR 'microbiome transfers, intestinal' OR 'transfer, intestinal microbiome' OR 'transfers, intestinal microbiome' OR 'fecal microbiota transfer' OR 'fecal microbiota transfers' OR 'microbiota transfer, fecal' OR 'microbiota transfers, fecal' OR 'transfer, fecal microbiota' OR 'transfers, fecal microbiota' OR 'fecal transplantation' OR 'fecal transplantations' OR 'transplantation, fecal' OR 'transplantations, fecal' OR 'vancomycin'/exp OR 'vancomycin' OR 'vancomycin hydrochloride' OR 'hydrochloride, vancomycin' OR 'vancomycin sulfate' OR 'sulfate, vancomycin' OR 'vancomycin-ratiopharm' OR 'vancomycin hexal' OR 'vancomycine dakota' OR 'ab-vancomycin' OR 'vanco azupharma' OR 'diatracin' OR 'vanco-cell' OR 'vanco-saar' OR 'vancocin' OR 'vancocin hcl' OR 'vancomycin lilly' OR 'vancocine' OR 'vancomicina abbott' OR 'vancomicina chiesi' OR 'vancomicina combino phar' OR 'vancomicina norman' OR 'fidaxomicin'/exp OR 'fidaxomicin' OR 'lipiarmycin a3' OR 'lipiarmycin' OR 'tiacumicin b' OR 'par 101' OR 'par101' OR 'par-101' OR 'dificid' OR 'lipiarmycin b4' OR 'tiacumicin c' OR 'lipiarmycin b' OR 'lipiarmycin a4' OR 'lipiarmycin b3c') |
| **Web of Science** | For **Web of Science**, the search used was:  #1:ALL=(‘Clostridioides difficile’ OR ‘Clostridium difficile’ OR ‘Clostridium Infections’ OR ‘Clostridium Infection’ OR ‘Infection, Clostridium’ OR ‘Infections, Clostridium’ OR ‘Clostridioides Infections’ OR ‘Clostridioides Infection’ OR ‘Clostridium difficile Infections’)  #2:ALL=(‘Fecal Microbiota Transplantation’ OR ‘Fecal Microbiota Transplantations’ OR ‘Microbiota Transplantation, Fecal’ OR ‘Microbiota Transplantations, Fecal’ OR ‘Transplantation, Fecal Microbiota’ OR ‘Transplantations, Fecal Microbiota’)  #3:ALL=(‘Vancomycin’ OR ‘Vancomycin Hydrochloride’ OR ‘Hydrochloride, Vancomycin’ OR ‘Vancomycin Sulfate’)  #4:ALL=(‘fidaxomicin’ OR ‘Lipiarmycin A3’ OR ‘Lipiarmycin’ OR ‘Tiacumicin B’ OR ‘PAR 101’ OR ‘PAR101’ OR ‘PAR-101’)  #5:#2 OR #3 OR #4  #6:#5 AND #1 |
| **Cochrane** | cFor **Cochrane**, the search used was:  #1 'clostridium difficile infection' OR 'Clostridioides perfringens Food Poisoning' OR 'Clostridium perfringens Food Poisoning' OR ‘Infection, Clostridium difficile’ OR 'Clostridium difficile Infections' OR 'Clostridium difficile Infection'OR 'Infection, Clostridioides difficile' OR 'Clostridioides difficile Infection' OR 'Clostridioides Infections' OR 'Clostridioides Infection' OR 'Infections, Clostridium' OR 'Clostridium Infection' OR 'Infection, Clostridium' OR 'Infection, Clostridium sordellii' OR 'Clostridioides sordellii Infection' OR 'Clostridium sordellii Infections' OR 'Infection, Clostridioides sordellii' OR 'Clostridium sordellii Infection' OR 'Clostridioides perfringens Infection' OR 'Clostridium perfringens Infection' OR 'Clostridioides perfringens Infections' OR 'Clostridium perfringens Infections'  #2 'Fecal Microbiota Transplantation' OR 'Transfer, Intestinal Microbiome' OR 'Microbiome Transplantations, Intestinal' OR 'Feces Infusion, Donor' OR 'Microbiome Transplantation, Intestinal' OR 'Intestinal Microbiota Transplant' OR 'Fecal Microbiota Transplants' OR 'Transplant, Intestinal Microbiome' OR 'Transplantations, Fecal Microbiota' OR 'Microbiota Transfers, Fecal' OR 'Infusion, Donor Feces' OR 'Fecal Transplant' OR 'Intestinal Microbiota Transplants' OR 'Fecal Microbiota Transfers' OR 'Transplantation, Fecal' OR 'Microbiota Transplants, Intestinal'  #3 'Vancomycin' OR 'VANCO-cell' OR 'AB-Vancomycin' OR 'Vancomycin-ratiopharm' OR 'Diatracin' OR 'Hydrochloride, Vancomycin' OR 'Vancomycin Hydrochloride' OR 'Vanco-saar' OR 'Vanco Azupharma' OR 'Vancocin HCl' OR 'Vancomycin Lilly' OR 'Vancocin' OR 'Vancocine' OR 'Vancomycin Hexal' OR 'Vancomicina Abbott' OR 'Sulfate, Vancomycin' OR 'Vancomycin Sulfate' OR 'Vancomycine Dakota' OR 'Vancomicina Chiesi' OR 'Vancomicina Combino Phar' OR 'Vancomicina Norman'  #4 'fidaxomicin' OR 'Lipiarmycin A3' OR 'Lipiarmycin' OR 'Tiacumicin B' OR 'Dificid; Lipiarmycin A4' OR 'Tiacumicin C' OR 'Lipiarmycin B4' OR 'Lipiarmycin B3' OR 'Lipiarmycin B' OR 'PAR101' OR 'PAR-101' OR 'PAR 101'  #5 #2 OR #3 OR #4  #6 #1 AND #5 |
| **Registered protocol** | |
| The protocol was registered at *https://www.crd.york.ac.uk/prospero/display_record.php?ID=CRD42021293294* | |

**Supplementary Table 4. Certainty of the evidence**

**Assessment of quality based on GRADE system**

|  | A VS B | AVS C | A-D | A-E | A-F | A-G | A-H | A-I | A-J | B-C |
| --- | --- | --- | --- | --- | --- | --- | --- | --- | --- | --- |
| Study limitations | ↓ | ↓ | ↓ | ↓ | ↓ | ↓ | ↓ | ↓ | ↓ | ↓ |
| Indirectness | ↓ | ↓ | ↓ | ↓ | ↓ | ↓ | ↓ | ↓ | ↓ | ↓ |
| Inconsistency | - | - | - | - | - | - | - | - | - | - |
| Imprecision | ↓ | - | ↓ | ↓ | ↓ | - | - | - | - | ↓ |
| Publication bias | - | - | - | - | - | - | - | - | - | - |
| GRADE | ⊕○○○ | ⊕⊕○○ | ⊕○○○ | ⊕○○○ | ⊕○○○ | ⊕⊕○○ | ⊕⊕○○ | ⊕⊕○○ | ⊕⊕○○ | ⊕○○○ |

|  | B-D | B-E | B-F | B-G | B-H | B-I | B-J | C-D | C-E | C-F |
| --- | --- | --- | --- | --- | --- | --- | --- | --- | --- | --- |
| Study limitations | ↓ | ↓ | ↓ | ↓ | ↓ | ↓ | ↓ | ↓ | ↓ | ↓ |
| Indirectness | ↓ | ↓ | ↓ | ↓ | ↓ | ↓ | ↓ | ↓ | ↓ | ↓ |
| Inconsistency | - | - | - | - | - | - | - | - | - | - |
| Imprecision | ↓ | - | - | - | ↓ | - | - | ↓ | ↓ | ↓ |
| Publication bias | - | - | - | - | - | - | - | - | - | - |
| GRADE | ⊕○○○ | ⊕⊕○○ | ⊕⊕○○ | ⊕⊕○○ | ⊕○○○ | ⊕⊕○○ | ⊕⊕○○ | ⊕○○○ | ⊕○○○ | ⊕○○○ |

|  | C-G | C-H | C-I | C-J | D-E | D-F | D-G | D-H | D-I | D-J |
| --- | --- | --- | --- | --- | --- | --- | --- | --- | --- | --- |
| Study limitations | ↓ | ↓ | ↓ | ↓ | ↓ | ↓ | ↓ | ↓ | ↓ | ↓ |
| Indirectness | ↓ | ↓ | ↓ | ↓ | ↓ | ↓ | ↓ | ↓ | ↓ | ↓ |
| Inconsistency | - | - | - | - | - | - | - | - | - | - |
| Imprecision | ↓ | ↓ | ↓ | ↓ | ↓ | ↓ | ↓ | ↓ | ↓ | ↓ |
| Publication bias | - | - | - | - | - | - | - | - | - | - |
| GRADE | ⊕○○○ | ⊕○○○ | ⊕○○○ | ⊕○○○ | ⊕○○○ | ⊕○○○ | ⊕○○○ | ⊕○○○ | ⊕○○○ | ⊕○○○ |

|  | E-F | E-G | E-H | E-I | E-J | F-G | F-H | F-I | F-J | G-H |
| --- | --- | --- | --- | --- | --- | --- | --- | --- | --- | --- |
| Study limitations | ↓ | ↓ | ↓ | ↓ | ↓ | ↓ | - | - | - | ↓ |
| Indirectness | ↓ | ↓ | ↓ | ↓ | ↓ | ↓ | ↓ | ↓ | ↓ | ↓ |
| Inconsistency | - | - | - | - | - | - | - | - | - | - |
| Imprecision | ↓ | ↓ | ↓ | ↓ | ↓ | ↓ | - | - | - | ↓ |
| Publication bias | - | - | - | - | - | - | - | - | - | - |
| GRADE | ⊕○○○ | ⊕○○○ | ⊕○○○ | ⊕○○○ | ⊕○○○ | ⊕○○○ | ⊕⊕⊕○ | ⊕⊕⊕○ | ⊕⊕⊕○ | ⊕○○○ |

|  | G-I | G-J | H-I | H-J | I-J |
| --- | --- | --- | --- | --- | --- |
| Study limitations | ↓ | ↓ | - | - | - |
| Indirectness | ↓ | ↓ | ↓ | ↓ | ↓ |
| Inconsistency | - | - | - | - | - |
| Imprecision | ↓ | ↓ | ↓ | ↓ | ↓ |
| Publication bias | - | - | - | - | - |
| GRADE | ⊕○○○ | ⊕○○○ | ⊕⊕○○ | ⊕⊕○○ | ⊕⊕○○ |

⊕⊕⊕○middle risk of evidence

⊕⊕○○low risk of evidence

⊕○○○very low risk of evidence

The study limitations were assessed based on the risk of bias assessment.

The indirectness was assessed by comparison of effect modifiers. We downgraded the quality of evidence of each one due to a lack of information about effect modifiers.

The inconsistency was determined based on 95%Prl of the OR. We downgraded the quality of evidence if their 95%Prl of the OR contained the clinical cutoff threshold.

The imprecision was determined by 95%Crl of the OR. We downgraded the quality of evidence if their 95%Crl of the OR contained the clinical cutoff threshold.

A funnel plot was created to assess publication bias.

**Supplementary Figure 1. Bias of graph**

**
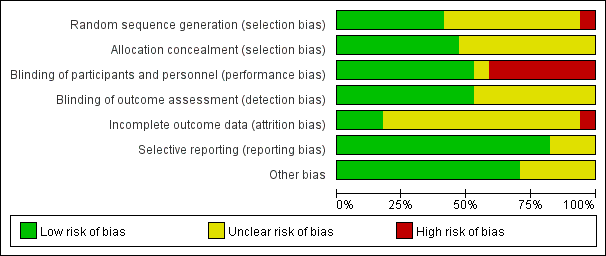
**

**Supplementary Figure 2. Bias of summary**

**
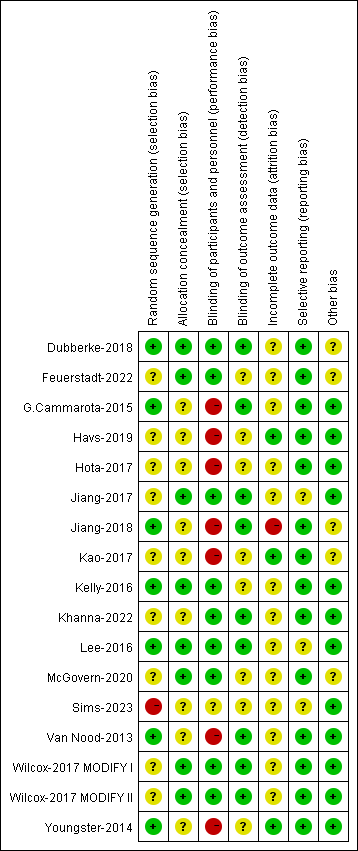
**

**Supplementary Figure 3. Contribution plot for the comparisons network.**

**
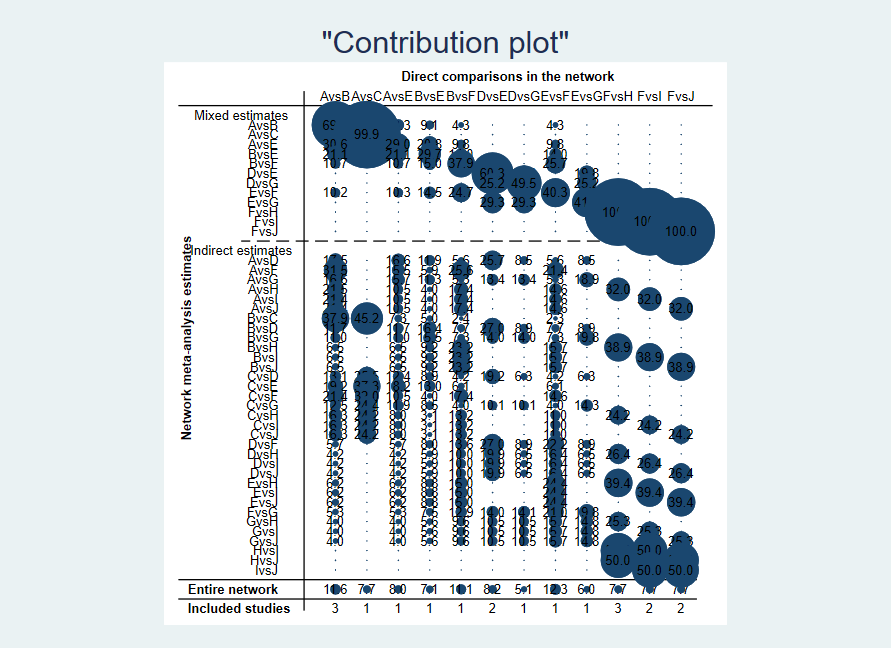
**

The numbers represent the percentage contribution of the column showing direct comparisons to the row defining network meta-analysis estimates. Treatment labels: A: FMT by LGI; B: FMT by UGI; C: AFMT; D: Vancomycin +FMT; E: Vancomycin; F: Placebo; G: fidaxomicin；H: SER109；I: RBX2660; J: Monoclonal antibody.

**Supplementary Figure 4. The forest plots of 28 pairwise comparisons and predicted intervals showed no difference in efficacy between the random-effects model and the fixed-effects model**

**
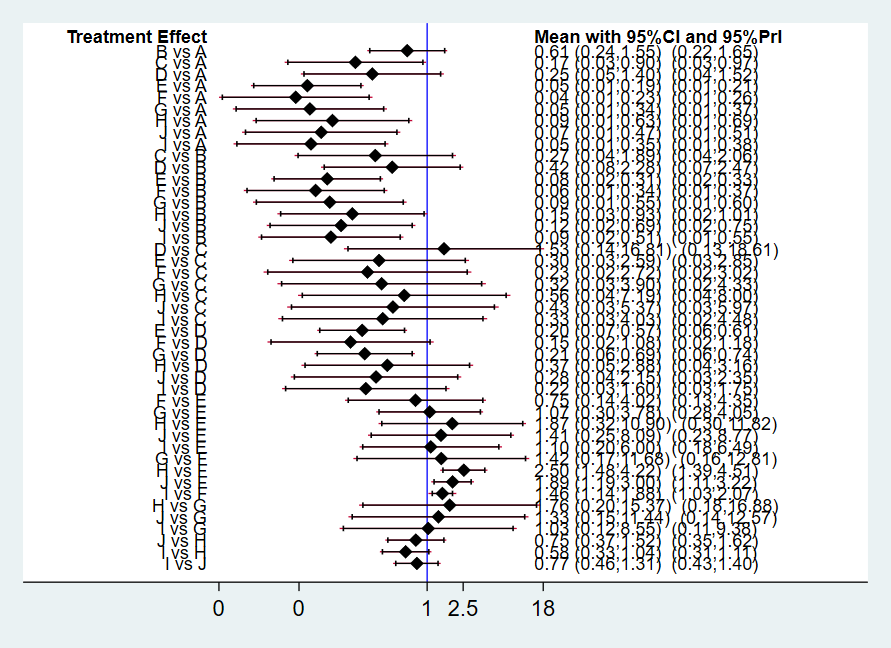
**

Treatment labels: A: FMT by LGI; B: FMT by UGI; C: AFMT; D: Vancomycin +FMT; E: Vancomycin; F: Placebo; G: fidaxomicin；H: SER109；I: RBX2660; J: Monoclonal antibody.

**Supplementary Figure 5. The assessment of inconsistency with a node-splitting method for all comparison loops.**

**
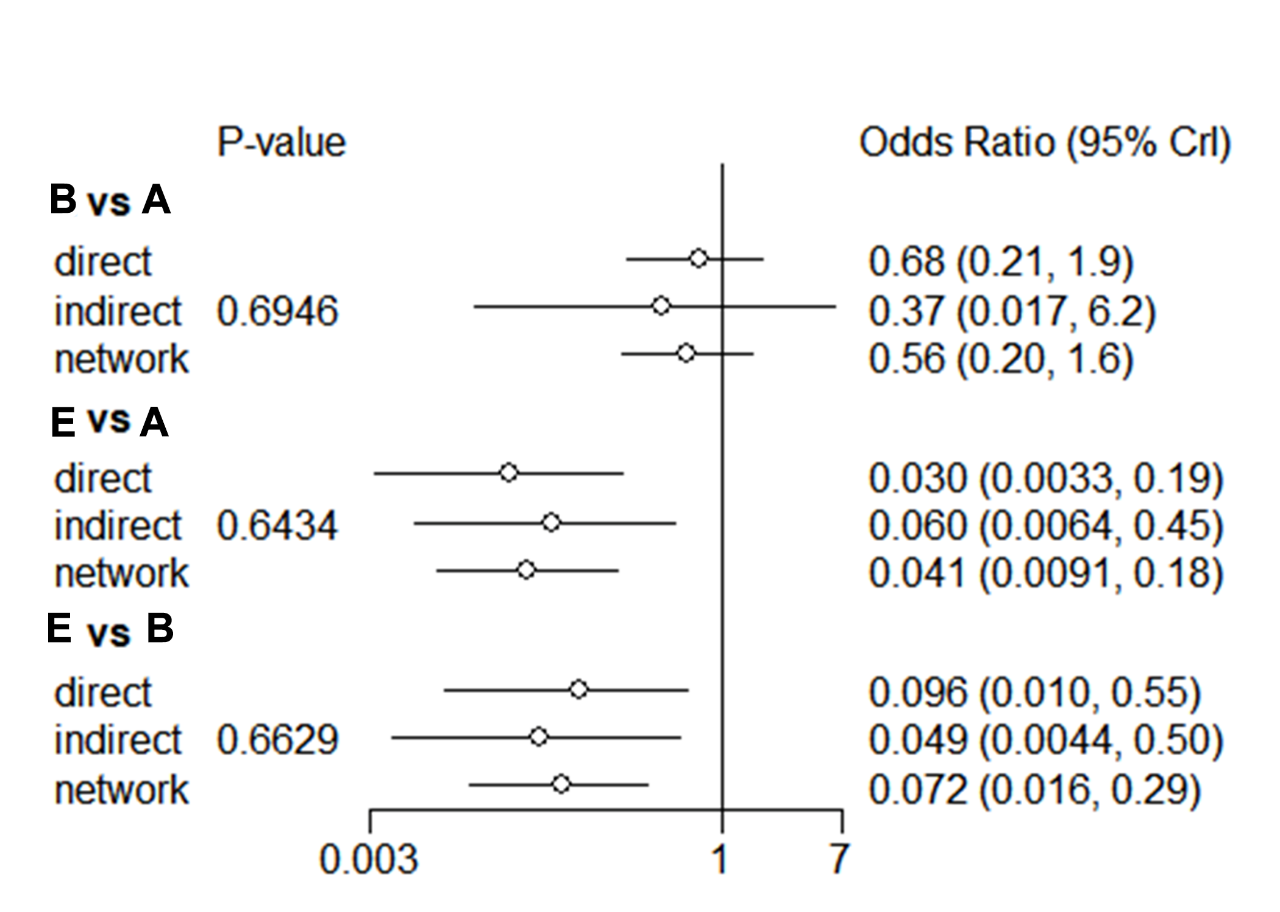
**

Treatment labels: A: FMT by LGI; B: FMT by UGI; C: AFMT; D: Vancomycin +FMT; E: Vancomycin; F: Placebo; G: fidaxomicin；H: SER109；I: RBX2660; J: Monoclonal antibody.

**Supplementary Figure 6. The assessment of heterogeneity.**

**
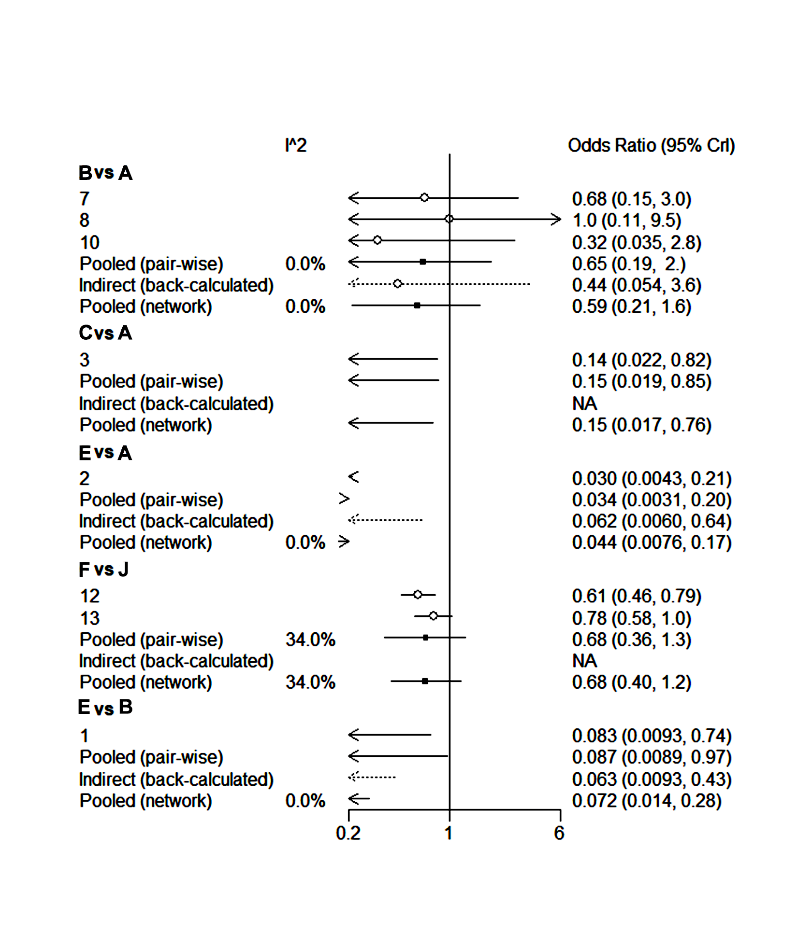
** **
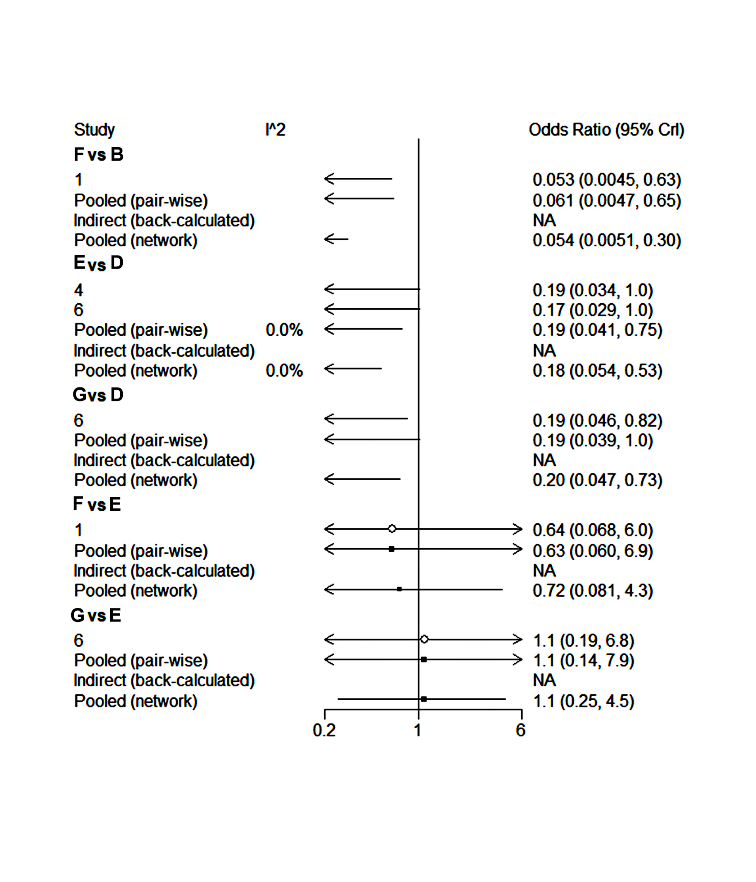
**
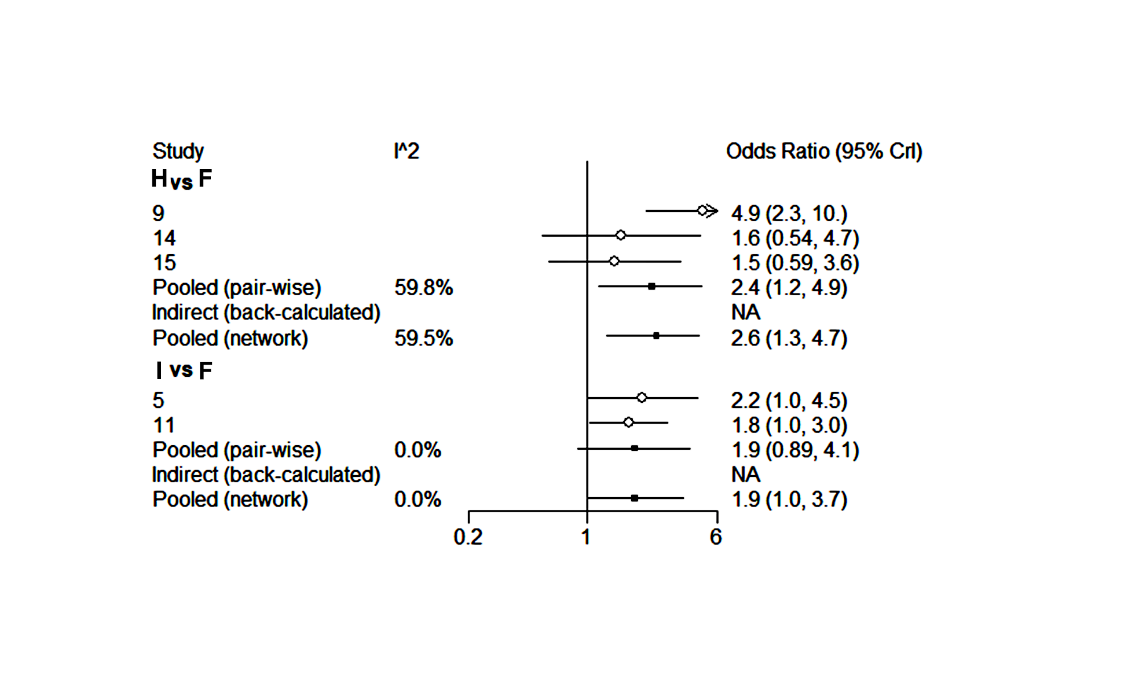


Treatment labels: A: FMT by LGI; B: FMT by UGI; C: AFMT; D: Vancomycin +FMT; E: Vancomycin; F: Placebo; G: fidaxomicin；H: SER109；I: RBX2660; J: Monoclonal antibody.

**Supplementary Figure 7. Forest plot**

**
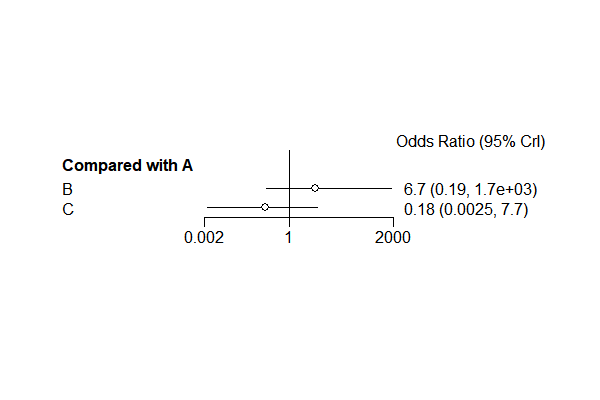
**

A: Frozen FMT; B: Fresh FMT; C: lyophilised
